# Utilizing ecological momentary assessment in nursing research

**DOI:** 10.4069/whn.2024.12.14.1

**Published:** 2024-12-30

**Authors:** Mona Choi

**Affiliations:** College of Nursing, Mo-Im Kim Nursing Research Institute, Yonsei University, Seoul, Korea

## Introduction

Ecological Momentary Assessment (EMA) refers to “a data collection method in which a study participant’s real-time self-reports of symptoms, behaviors, and other experiences are collected in their natural environment at or close to the time they occur, often via electronic devices such as smartphones or other handheld technologies” [1]. Although EMA was adopted as a Medical Subject Headings term in 2017 [1], its conceptual origins are much older. First proposed in the field of psychology by Larson and Csikszentmihalyi [2] in 1983, the experience sampling method is an umbrella term for a family of momentary assessment techniques that use signals to trigger data collection in daily life [2,3]. Later, in 1994, Stone and Shiffman published a related paper introducing the term “ecological momentary assessment” [4]. Additionally, there is a related term, “ambulatory assessment,” which emphasizes data collection in the participant’s natural environment [5]. When to capture a particular moment is a critical determinant in EMA. To date, data collection tools have evolved to include smartphone applications and wearable sensors, such as wrist-worn accelerometers [6-8]. These advances include ecological validity, which enables real-world data collection, and the ability to collect data repeatedly, which is advantageous for identifying dynamic behavioral patterns and temporal changes [5,9].

The purpose of this review is to explore the potential of EMA in nursing research. It examines the conceptual foundations of EMA, methodological approaches for practical implementation, and the challenges and emerging opportunities for its application in the field.

## Why ecological momentary assessment is needed in nursing research

Patient experiences, symptoms, behavioral changes, and emotional states are complex and multidimensional, which is a major issue in nursing. Collecting data through EMA is highly advantageous because it allows more precise data collection in a variety of domains. Furthermore, some EMA-related applications offer the ability to integrate weather data to simultaneously record the impact of weather on emotions or physical activity. This approach facilitates a deeper analysis by combining environmental factors with psychological and behavioral data. The potential to utilize physiological data in EMA is also expanding through integration with wearable devices. For example, combining EMA with devices that can continuously monitor physiological metrics such as heart rate and glucose levels has great potential for nursing research.

EMA has been utilized in a variety of nursing studies, including those involving patients, older adults in the community, and nursing students [7,10,11]. One study revealed dynamic patterns of mood and stress in moyamoya disease patients, identifying context-specific factors influencing psychological states at both individual and group levels, which informed potential targeted interventions [7]. Another study utilized EMA alongside machine learning algorithms, wearable devices, and diverse data collection methods to predict depression among older adults living alone, demonstrating high accuracy and offering a promising approach for early identification and monitoring of geriatric depression [10]. For the study with nursing students, a study used EMA to explore nursing students’ experiences of clinical activities during and after clinical placements with a focus on feelings of competence and challenge [11]. These studies highlight the ability of EMA to capture real-time data on complex psychosocial and psychiatric phenomena, such as mood, stress, depression, and learning experiences, by exploring momentary changes and individual differences that traditional retrospective methods often fail to detect. The potential applications of EMA in nursing research are expected to expand significantly in the future.

### Perspectives of ecological momentary assessment research

In exploring the perspectives of EMA research, four primary investigative domains emerge: analyzing individual differences, natural history observations, contextual associations, and temporal sequences [5,9,12]. First, EMA enables real-time analysis of individual differences, allowing researchers to quantitatively assess dynamic variations—such as changes in pain after interventions—without relying on retrospective recall. Second, the method facilitates natural history observations by tracking within-person changes and identifying specific trends in an individual’s daily life. Third, EMA provides a sophisticated approach to analyzing contextual associations, revealing how phenomena interact within temporal and environmental contexts, such as exploring correlations between specific settings (e.g., at a park, at work, etc.) and emotional states. Finally, EMA offers insights into temporal sequences by capturing causal relationships between events through a detailed evaluation of their antecedents and consequences, thereby providing a nuanced understanding of complex behavioral dynamics.

### Advantages of ecological momentary assessment

Repeatedly measuring everyday experiences and behaviors in real-time inherently reduces the errors and biases that can occur during recall. Receiving real-time data at a specific point in time attenuates the memory distortions that are common in research using surveys. In addition, this approach is highly reliable, as it allows the collection of more accurate and sensitive data than retrospective reporting, which can suffer from recall bias [9,12].

Another strength is the ability to measure daily fluctuations in psychological and behavioral traits. This allows the researcher to capture subtle changes in participants and detect changes in psychological state or behavior with greater precision. Another important advantage is the ability to capture real-world experiences more vividly. It also provides an opportunity to compare traditional self-report methods with objective data collection methods. For example, using wearables to collect data has the additional advantage of allowing simultaneous comparative analysis of subjective and objective data [9].

## Methodological strategies for practical considerations

### Optimizing data collection

The data collection strategy can vary depending on the objectives of the research and the availability of resources.

#### Duration, frequency, and participant burden

Determining the duration and frequency of data collection is a critical step in research design [9]. The selection is guided by the research topic, theoretical background, phenomenon of interest, and specific variables under investigation. The total data collection frequency is calculated by multiplying the study duration by the daily assessment frequency. For instance, a study examining within-person associations between environmental factors (e.g., weather) and personal factors (e.g., affect) with physical activity collected data five times a day for 7 days, yielding 35 EMA collection points per participant [13]. A systematic review of EMA research on stress and mood revealed considerable variability, with study durations ranging from 7 to 336 days and prompt frequencies varying from 1 to 10 daily assessments [14].

A key challenge in study design is collecting data efficiently while minimizing the burden on participants [9]. A study showed that perceived burden can significantly impact protocol adherence, with nonadherence on the current or previous day correlating with increased participant strain [15]. Therefore, researchers must carefully balance the desire for data richness with the potential risk of participant fatigue.

#### Notification intervals

In EMA research, notification intervals are crucial for data collection, and several approaches are used [9,12]. Random notifications are sent unpredictably within a specified time frame, such as between 8 a.m. and 8 p.m. Semi-random notifications involve a predetermined frequency (e.g., three times daily) with randomized timing within designated time frames such as morning, afternoon, and evening. Fixed-interval notifications occur at consistent, predetermined times throughout the day. Fixed-preference notifications are scheduled according to participant- or researcher-selected time slots (e.g., 9:00 a.m., 1:00 p.m., 6:00 p.m.). Self-initiated or event-based notifications are triggered by specific events or user actions rather than scheduled intervals. For example, participants with chronic pain can log their pain level immediately after experiencing a significant pain episode, capturing the precise moment and intensity of their physical experience.

#### Signaling method

Another important factor in EMA design is the signaling method. This can be a combination of event-based and time-based.

##### 1) Event-based designs

Event-based designs focus on monitoring specific, predefined events in clinical and research settings. These events are established based on specific triggers, such as panic attacks, social interactions lasting more than 10 minutes, or medication use. Triggers are often collected in the form of self-reports. The key to event-based designs is clearly defining what constitutes an event. For example, when collecting physical activity data, it is crucial to clearly specify what qualifies as “exercise” to ensure consistent and accurate data collection and analysis. Additionally, some events may vary in intensity (e.g., severe pain, intense cravings to smoke), so careful consideration is required regarding how these events are recorded and analyzed [9].

##### 2) Time-based designs

Time-based designs are suitable when specific events are challenging to capture or when the variable of interest is continuous. For example, variables such as mood or pain may be difficult to isolate into discrete episodes, making time-based designs more appropriate for systematic data collection. These designs often employ various devices (e.g., actigraphy) to collect and monitor data continuously. The frequency, interval, and timing of data collection can be adjusted to fit the research objectives. However, even in time-based designs, there is a risk of data bias if the timing of data collection consistently overlaps with the specific emotional states of the participant. For instance, if data are always collected at 6 p.m., and the participant typically feels stressed during that time, the data may overrepresent stress and fail to capture the participant’s emotional states during other, potentially less stressful, parts of the day. This could lead to an incomplete or skewed understanding of their overall emotional experience. Therefore, such designs need to account for this potential bias to maintain data validity [9].

#### Operating system and platform selection

The choice of mobile operating system is an important consideration in EMA data collection when using a mobile application [16]. As of 2024, Android has 72% of the global market share. In Korea, more than two-thirds of smartphone users reported using Android phones [17]. Therefore, researchers should consider this platform based on their study population. However, the iOS platform is also important to consider for certain target groups, such as younger generations.

While it is possible to develop a custom application to collect EMA data, high development costs can be a significant constraint. As a practical alternative, researchers should consider utilizing existing mobile platforms such as online survey tools, social network services, and health apps developed and distributed by companies to reduce development and maintenance costs.

### The importance of the completion rate

The completion or compliance rate is a critical factor in EMA studies [9,12,18-20]. Systematic reviews have reported varying levels of compliance rates across different populations. A review evaluating mood and stress in adult patients found compliance rates ranging from 65% to 89% [14], while another systematic review of EMA studies on older adults reported a wider range of 36% to 98%, with a pooled rate of 86.4% [21]. The completion rate is intimately linked to the reliability and analytic utility of collected data, serving as a key indicator of study results’ validity. Therefore, researchers must develop strategic approaches to increase completion rates during study design and data collection processes.

Compliance can be challenging for participants, as it requires recording data at predetermined times within their daily lives. Moreover, the type and pattern of missing data during collection can significantly impact study analysis. Missing data and compliance are crucial indicators for ensuring data collection reliability, necessitating a strategic design to optimize participant engagement [9].

A study of adult patients with moyamoya disease, which collected data four times daily over 1 week, observed a significant decline in compliance by the fifth day, with an average compliance rate of approximately 70% [22]. Compliance was influenced by EMA design characteristics, including study duration, frequency of response reminders, and the length of assessment items. Studies reported that younger participants, longer durations, higher response frequencies, and lengthier response requirements have generally reported lower completion rates [18-20], although some studies found mixed or no differences [23,24]. These findings emphasize the importance of thoughtful study design and the implementation of motivational strategies to sustain participant engagement. For instance, providing regular feedback through phone calls or text messages has proven effective in maintaining motivation and involvement. Such targeted interventions are essential for the successful execution of EMA studies, ensuring high data quality and participant commitment.

### Approaches to data analysis

The analysis of EMA data presents unique challenges and opportunities due to the vast volume of information collected compared to traditional cross-sectional studies. In EMA studies, each individual participant provides numerous self-reports throughout the study period influenced by the study’s sampling design (e.g., daily assessment frequency) and overall study duration. The resulting dense, longitudinal data enables unprecedented insights into individual psychological and behavioral fluctuations, while simultaneously necessitating advanced statistical techniques to navigate and meaningfully interpret the intricate patterns of within-person variability and temporal dynamics [9,12].

These repeated measures data are also termed intensive longitudinal data [25]. Since EMA studies collect data at frequent time intervals, repeated measures analysis is typically employed. A primary analytical approach is the linear mixed model, which effectively addresses both within-person variability (random effects) and between-person variability (fixed effects). Specifically, the linear mixed model or generalized linear mixed model has emerged as a robust method for analyzing non-normally distributed data (e.g., binary variables) with fixed random effects [26]. EMA excels at capturing within-person and between-person variability, making multilevel models particularly suitable for simultaneously analyzing individual and group-level effects [27,28].

The potential for one time point to influence subsequent measurements introduces an autocorrelation problem that requires careful analysis. Missing data can occur randomly or non-randomly, and studies have proposed varying rates of data loss and inclusion criteria [27]. The richness of EMA data is enhanced when contextualized by participant-specific factors, ultimately improving the utilization of research results. Moreover, tutorial resources are available for EMA data analysis [28,29].

## Challenges and future directions

As mentioned earlier, frequent notifications during EMA data collection can induce participant fatigue, directly impacting compliance rates. Optimizing notification frequency and minimizing participant burden represents a critical methodological challenge. The intricate nature of EMA data necessitates sophisticated multilevel analysis techniques and advanced data processing capabilities. Moreover, EMA is highly technology-dependent, making app design and technical implementation crucial considerations in study design.

An important methodological concern is the potential for the EMA assessment process itself to influence participants’ behavior or experiences. Researchers must be cognizant that the act of requesting data may trigger reactivity, and this factor should be meticulously addressed during the study’s design phase.

EMA is increasingly being integrated with wearable technology, with ecological momentary intervention emerging as a promising approach for delivering personalized interventions. While current applications predominantly focus on assessment, the potential for timely, context-specific interventions is rapidly expanding.

The integration of EMA with telemedicine offers unprecedented opportunities for remote patient monitoring and real-time interventions. This integration can significantly boost patient engagement, as individuals are more likely to be motivated when their symptoms are directly connected to healthcare providers. Developing strategies to maintain and improve such engagement represents a promising avenue for future EMA research.

Furthermore, EMA’s capacity to collect high-density data positions it as an ideal methodology for big data analytics and data science, potentially broadening the horizons of nursing research and healthcare innovation.

## Conclusion

EMA represents a transformative methodology in nursing research, providing real-time, ecologically valid insights into complex health phenomena. The integration of EMA with wearable technology and big data analytics establishes it as a valuable approach for future innovations in nursing. Continued exploration of its capabilities is essential for developing timely, context-specific interventions that advance nursing research and practice.
